# STANDARDIZATION OF THE WHITE TEST IN OPEN LIVER RESECTION: TOWARD NEAR-ZERO CLINICALLY SIGNIFICANT BILE LEAKAGE

**DOI:** 10.1590/0102-6720202500007e1876

**Published:** 2025-04-07

**Authors:** Gabriel LAZZAROTTO-DA-SILVA, Tomaz de Jesus Maria GREZZANA-FIILHO, Ian LEIPNITZ, Flávia Heinz FEIER, Pablo Duarte RODRIGUES, Celina Pereira HALLAL, Marcio Fernandes CHEDID, Cleber Rosito Pinto KRUEL

**Affiliations:** 1 Universidade Federal do Rio Grande do Sul, Hospital de Clínicas de Porto Alegre, Department of Liver Transplant and Hepatobiliary Surgery - Porto Alegre (RS), Brazil; 2 Universidade Federal do Rio Grande do Sul, Post-Graduate Program in Surgical Sciences - Porto Alegre (RS), Brazil.

**Keywords:** Biliary Fistula, Hepatectomy, Pancreatitis, Fístula Biliar, Hepatectomia, Pancreatite

## Abstract

**BACKGROUND:**

Biliary fistula is one of the most common complications after liver resection and is associated with significant morbidity and mortality. One of the methods used to evaluate biliary fistulas is the White test, which consists of injecting a lipid emulsion into the bile duct. However, no standard technique for performing the White test has been published.

**AIMS:**

The aim of this study was to standardize the technique for performing the White test in patients undergoing hepatectomies, with and without previous cholecystectomy, and to assess the preliminary results.

**METHODS:**

Patients over 18 years of age who were submitted to open hepatectomy were included in the study. The primary outcome was the rate of biliary fistula. Secondary outcomes were the incidence of acute pancreatitis and overall morbidity, measured by the Clavien-Dindo classification.

**RESULTS:**

The standard technique for the White test was performed on 17 patients. In total, three patients had previous cholecystectomy, and two had low insertion of the cystic duct, requiring cannulation of the hepatocholedochal duct. None of the patients developed clinically significant biliary leaks. Acute pancreatitis did not occur in any patient. One patient developed pneumonia requiring mechanical ventilation (Clavien-Dindo IV). All others had minor or no complications.

**CONCLUSIONS:**

The standardized technique for performing the White test suggests an appropriate strategy to maximize the detection of intraoperative biliary leaks.

## RESUMO

**RACIONAL:**

Fístula biliar é uma das complicações mais comuns após ressecção hepática e é associado a significativa morbimortalidade. Um dos métodos empregados para avaliar fístulas biliares é o *white test*, que consiste na injeção de uma emulsão lipídica na via biliar. No entanto, nenhuma técnica padrão para realização do *white test* foi publicada.

**OBJETIVOS:**

Padronização da técnica para realização do *white test* em pacientes submetidos a hepatectomias, com e sem colecistectomia prévia e os resultados preliminares.

**MÉTODOS:**

Paciente acima de 18 anos e submetidos a hepatectomia aberta foram incluídos na pesquisa. O desfecho primário foi a taxa de fístula biliar. Desfechos secundários foram a incidência de pancreatite aguda e morbidade geral, medida pela classificação de Clavien-Dindo.

**RESULTADOS:**

A técnica padrão para o *white test* foi realizada em 17 pacientes. Três pacientes tinham colecistectomia prévia e 2 tinham inserção baixa do ducto cístico, necessitando canulação do ducto hepatocolédoco. Nenhum dos pacientes desenvolveu fístula biliar clinicamente significativa. Pancreatite aguda não ocorreu em nenhum paciente. Um paciente desenvolveu pneumonia necessitando ventilação mecânica (Clavien-Dindo IV). Todos os outros tiveram complicações menores ou não tiveram complicações.

**CONCLUSÕES:**

A técnica padronizada para realização do *white test* sugere uma estratégia adequada para maximizar a detecção de vazamentos biliares no intraoperatório.

**Figure 3:**
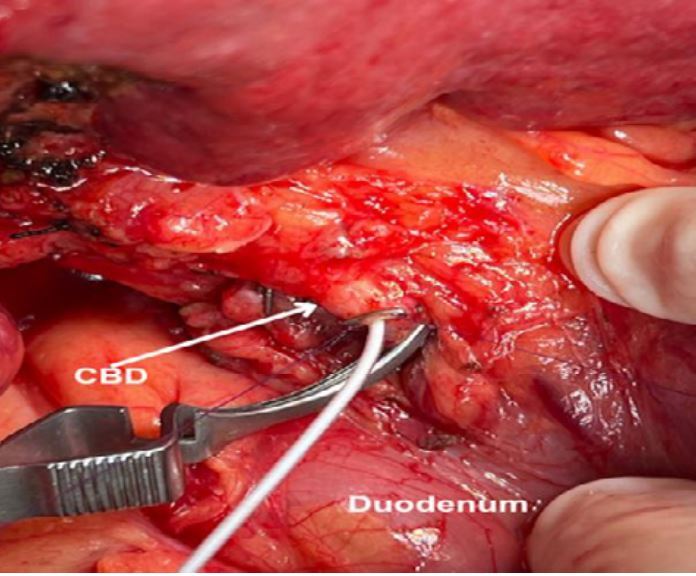
Cannulation of the common bile duct (CBD) in a case of non-anatomical resection of two lesions in segments VIII and IVa (not shown). Two 6-0 polydioxanone sutures were passed longitudinally along the CBD axis. The site of catheter insertion in the CBD was secured by a hemostatic clip.

**Central Message:**

Hepatic resection is the treatment of choice for most liver tumors. Although significant technical advancements have been achieved in the last decades, postoperative morbidity remains a significant issue. Bile leakage is one of the most common postoperative complications and is associated with high surgical morbidity and mortality.

**Perspectives:**

The standardized technique for the White test, which focuses on cystic duct or common bile duct cannulation with distal common bile duct clamping, seems a safe and adequate strategy to maximize intraoperative bile leak detection and prevent acute pancreatitis. Several methods have been developed to facilitate intraoperative identification of bile leaks. Despite the widespread adoption of the White test, the technique varies among specialized centers.

## INTRODUCTION

Hepatic resection is the treatment of choice for most liver tumors^17^. Although significant technical advancements have been achieved in the last decades, postoperative morbidity remains a significant issue^4^ ^,^ ^11^ ^,^ ^24^. Bile leakage, which occurs in 3-26% of patients, is one of the most common postoperative complications^8^ ^,^ ^9^ ^,^ ^14^ ^,^ ^20^ and is associated with high surgical morbidity and mortality^7^ ^,^ ^15^.

Several methods have been developed to facilitate intraoperative identification of bile leaks^10^ ^,^ ^14^ ^,^ ^24^. One of them, commonly referred to as the White test, consists of the injection of a sterile fat emulsion in the biliary tree^16^. Despite the widespread adoption of the White test, the technique varies among specialized centers. Additionally, a case series reported three patients who presented with acute pancreatitis associated with the White test, raising concerns about its safety^5^. To our knowledge, no standard technique for a bile leak test has ever been published before.

The objectives were to systematize a standard technique for performing the White test in patients submitted to hepatectomy, with and without previous cholecystectomy, and report preliminary results.

## METHODS

This prospective study enrolled all consecutive patients >18 years old submitted to open liver resection for any diseases. Patients who needed resection of the extrahepatic biliary tree and bilioenteric anastomosis were excluded.

### Surgical technique

After opening the abdominal wall, the abdominal cavity is carefully examined to look for metastatic disease. In patients without previous cholecystectomy, cholecystectomy is performed in all cases as the first surgical step. The cystic duct is ligated with 2-0 silk suture, left long to facilitate later identification of the cystic duct stump.

After cholecystectomy, hilar dissection and intrafascial ligation of the right portal vein and right hepatic artery or the left portal vein and left hepatic artery are performed in cases of anatomical right and left hepatectomy, respectively. No hilar dissection is carried out in cases of non-anatomic resection.

**Figure 1:**
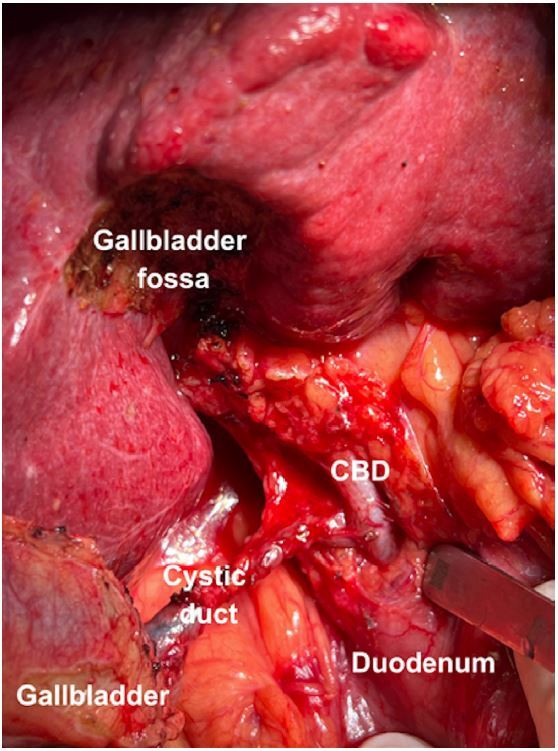
Low insertion of the cystic duct in the common bile duct (CBD). The gallbladder was detached from the gallbladder fossa and the cystic duct was dissected until the confluence with the CBD was identified.

Liver mobilization is performed as appropriate to expose the hepatic segment or section to be resected. Parenchymal transection is performed using the SONOPET^®^ ultrasonic aspirator. Intrahepatic glissonian pedicles and hepatic vein branches are ligated with 2-0 or 3-0 silk, 4-0 or 5-0 polypropylene, and/or hemostatic clips, depending on vessel diameter. Upon completion of hepatic resection and hemostasis, preparation for the White test is carried out as follows:

1. Patients without previous cholecystectomy: The cystic duct stump is identified and dissected carefully until its confluence with the common hepatic duct is made visible. Additionally, the distal common bile duct (CBD) is exposed from adjacent connective tissue, enough to allow placement of a bulldog clamp. Whenever a bulldog clamp can be safely placed below the confluence of the cystic duct and common hepatic duct, the cystic duct stump is chosen for cannulation. On the other hand, when the insertion of the cystic duct is too low to allow the clamp to be placed below it (Figure 1), the common hepatic duct is chosen for cannulation.

2. Patients with previous cholecystectomy: Minimal dissection of the gallbladder fossa is performed to search for the cystic duct stump from previous surgery. If the cystic duct stump is easily found and safely dissected away from the main biliary tree, the stump is opened and cannulated. However, when the cystic duct stump cannot be found with minimal dissection, the CBD is chosen for cannulation.

### Cannulation of the biliary tract for the White test

**Figure 2:**
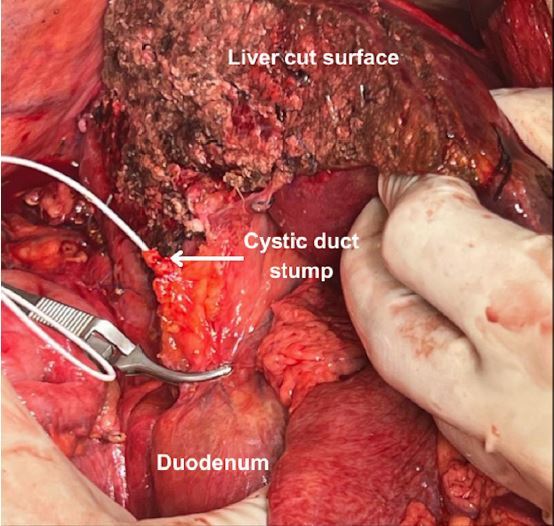
Cannulation of the cystic duct stump in a case of right hepatectomy.

- Cannulation of the cystic duct: The distal CBD is clamped with a bulldog clamp. A 6 French rubber catheter is inserted in the cystic duct and advanced 2-3 cm. A 20 mL syringe filled with normal saline is connected to it. The surgeon then places a hemostatic clip around the cystic duct stump to secure the catheter and progressively tightens the clip while the assistant injects normal saline (Figure 2). As soon as significant resistance to flow is identified by the assistant, he or she communicates it to the surgeon, who interrupts the tightening of the clip around the cystic duct. More saline is gently injected to ensure there is no leakage around the catheter.

- Cannulation of the common hepatic duct: Two parallel longitudinal (along the CBD axis) sutures of 6-0 polydioxanone are placed about 3-4 mm apart. The sutures are gently pulled to stretch the anterior wall of the CBD. A 2-3 mm choledocotomy is made using a number 11 scalpel blade. A 4 French rubber catheter is inserted through the choledocotomy site and advanced 2-3 cm into the proximal hepatic duct. A 20 mL syringe filled with normal saline is connected to the catheter. To secure the catheter and seal its entrance in the CBD, the sutures are again gently pulled, and a hemostatic clip is placed around the catheter entry site (Figure 3). The surgeon progressively tightens the clip while the assistant injects normal saline as described above. The distal CBD is clamped with a bulldog.

**Figure 3:**
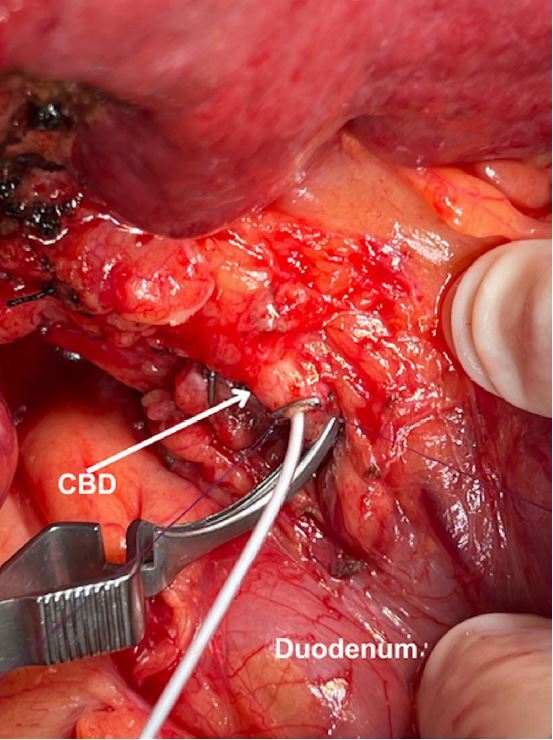
Cannulation of the common bile duct in a case of non-anatomical resection of two lesions in segment VIII and IVa (not shown). Two 6-0 polydioxanone sutures were passed longitudinally along the common bile duct axis. The site of catheter insertion in the common bile duct was secured by a hemostatic clip.

### Injection of fat emulsion

A syringe containing the solution for the White test is attached to the catheter. The solution is prepared with 100 mL of 20% fat emulsion (Lipofundin^®^) and 300 mL of normal saline, resulting in 5% fat emulsion. The assistant injects 30 mL of the solution in 1 min while the surgeon carefully examines the liver cut surface. A sign of adequate cannulation is the whitish discoloration of the extrahepatic biliary tree. All bile leaks are sewn with 3-0 or 4-0 polypropylene sutures and/or hemostatic clips. Whenever a leak is detected, an additional 5 mL of the solution is injected. The test is repeated with 5 mL of the solution until no leak can be observed.

### End of the test

Upon completion of the test, the hemostatic clip is removed, and the catheter is pulled out of the cystic duct or common hepatic duct while the distal bulldog clamp is left in place. This is done to prevent high endobiliary pressure from being transmitted distally, which could lead to high-pressure fat emulsion reflux into the pancreatic duct. After the biliary tree is emptied of the fat emulsion and bile starts leaking from the cystic duct stump or choledocotomy site, the bulldog clamp is removed. When the cystic duct was cannulated, the cystic duct stump is closed with two hemostatic clips. Conversely, if the CBD was cannulated, the choledocotomy site was closed with a running suture using the previously placed 6-0 polydioxanone suture. A 24 French tubular drain (Blake^®^) is left in contact with the hepatic cut surface.

### Postoperative protocol

On postoperative day 1, liver function tests including serum total bilirubin are measured. Serum amylase and lipase are also measured. The drain fluid is collected for total bilirubin measurement. If the bilirubin concentration is below three times as high as the serum bilirubin, the drain is removed on postoperative day 1 or 2 at the surgeon’s discretion. When the drain fluid bilirubin concentration is three times greater than serum bilirubin, the drain is left in place and measured again on postoperative day 3. If drain fluid bilirubin concentration remains high, the same process is repeated every other day.

### Outcomes

The primary outcome was the occurrence of bile leakage, which was defined as fluid with bilirubin concentration at least three times greater than serum bilirubin on postoperative day 3 or later or the need for radiologic intervention because of biliary collections or relaparotomy resulting from bile peritonitis^12^. Additionally, bile leakage was graded as follows: grade A: bile leakage requiring no or little change in patients’ clinical management; grade B: bile leakage requiring a change in patients’ clinical management (e.g., additional diagnostic or interventional procedures) but manageable without relaparotomy, or a grade A bile leakage lasting for >1 week; grade C: bile leakage requiring relaparotomy.

Secondary outcomes were the occurrence of postoperative acute pancreatitis as defined by the revised Atlanta definition^2^ and postoperative complications, which were graded according to the Clavien-Dindo classification^6^. Minor complications were defined as Clavien-Dindo I or II, whereas Clavien-Dindo III or higher was considered a major complication.

The research was approved by the Ethics Committee of the Institution (number 2024-0004).

## RESULTS

This study included 18 patients undergoing open liver resection without bilioenteric anastomosis at the authors’ institution. One patient was excluded due to extensive adhesions from previous open liver resection and cholecystectomy making hilar dissection for CBD cannulation unsafe. The remaining 17 patients were included in the study. Individual case descriptions are shown in Table 1. The median age was 62 (range 29-73 years). Notably, 7 patients were male and 11 were female. Indication for liver resection was hepatocellular carcinoma in eight patients and colorectal liver metastasis in five patients. Other indications were intrahepatic cholangiocarcinoma (1 case), focal nodular hyperplasia, which was suspected to be cutaneous melanoma on preoperative imaging (1 case), clear cell liver carcinoma (1 case), and living donor hepatectomy (1 case). Major liver resection (three or more Couinaud segments) was performed in six patients, of which three were right hepatectomies, two were left hepatectomies, and one was a central hepatectomy along with two additional non-anatomical resections. Minor liver resection was performed in 11 patients, of whom four were left lateral sectionectomies, one was a right posterior sectionectomy, and six were non-anatomical minor resections.

**Table 1:** Cases records.

No	Age	Indication	Type of liver resection	Previous cholecystectomy	Site of biliary cannulation	Intraoperative leak sites	Bile leakage grade	Drain length of stay (days)	Complications (Clavien-Dindo)
1	63	HCC	Right	No	Cystic duct	1	0	7	0
2	29	LDLT	Left lateral	No	Cystic duct	0	0	2	0
3	55	HCC	Non-anatomic	Yes	CBD	0	0	3	I
4	51	Clear cell carcinoma	Right	No	Cystic duct	1	0	2	I
5	63	HCC	Right	No	CBD	2	0	3	I
6	66	HCC	Left lateral	No	Cystic duct	1	0	2	0
7	70	ICC	Right posterior	No	Cystic duct	0	A	6	I
8	73	HCC	Left	Yes	CBD	2	0	2	0
9	73	HCC	Non-anatomic	Yes	CBD	0	0	1	IV
10	72	HCC	Left lateral	No	Cystic duct	0	0	2	I
11	41	CRLM	Non-anatomic	No	Cystic duct	0	0	2	0
12	34	FNH	Left lateral	No	Cystic duct	0	0	1	I
13	61	HCC	Left	No	Cystic duct	2	A	5	I
14	55	CRLM	Non-anatomic	No	Cystic duct	1	0	1	0
15	73	CRLM	Non-anatomic	No	Cystic duct	1	0	1	0
16	60	CRLM	Central	No	Cystic duct	2	0	2	I
17	63	CRLM	Non-anatomic	No	CBD	0	0	2	0

HCC: hepatocellular carcinoma; LDLT: living donor liver transplantation; ICC: intrahepatic cholangiocarcinoma; CRLM: colorectal liver metastasis; FNH: focal nodular hyperplasia; CBD: common bile duct.

### White test

Previous cholecystectomies had been performed on three patients, in whom the White test was performed via CBD cannulation. Additionally, the CBD was cannulated in two patients with low confluence of the cystic duct and CBD, precluding placement of a bulldog clamp below it. The White test was positive in 10 patients. Intraoperative bile leak occurred in only one site in six patients and in two sites in four patients.

### Postoperative outcomes

In total, two patients met the definition of grade A biliary leakage. Serum bilirubin and drain fluid bilirubin were, respectively, 0.5 and 1.8 mg/dL in one patient and 1 and 3.3 mg/dL in the other. Both patients remained asymptomatic, and their drains were removed on postoperative days 5 and 6, respectively. All other patients did not present biliary leakage, and all but one of those patients had their drains removed at or before postoperative day 3. Notably, one patient who did not develop biliary leakage had his drain left in place until postoperative day 7 owing to extensive diaphragmatic repair and risk of biliopleural fistula in case undrained bile leakage occurred. Only one patient had major complications (pneumonia necessitating mechanical ventilation), which was classified as Clavien-Dindo IV. A total of seven patients had no complications, and nine had minor complications (Clavien-Dindo I). Acute pancreatitis did not occur in any patient. One patient had asymptomatic amylase elevation (428 U/L) but normal lipase on postoperative day 1. The median length of stay was 6 days (range 3-73 days).

## DISCUSSION

Bile leakage is a significant complication after liver resection, increasing the risk of sepsis^4^ ^,^ ^14^, need for other procedures^3^, postoperative liver failure, and death^8^. Intraoperative bile leak testing not only decreases the incidence of postoperative bile leakage^22^ but also results in reduced postoperative morbidity and shorter length of stay^7^ ^,^ ^21^. Several substances have been proposed for performing the bile leak test, namely isotonic saline^10^, methylene blue^14^, and indocyanine green^23^. However, there are some disadvantages to all of them. While isotonic saline is transparent, making it difficult to identify small leaks, methylene blue and indocyanine green lead to staining of the liver cut surface, potentially masking additional leak sites. To minimize these deficiencies, Nadalin et al.^16^ developed the White test, which uses a lipid emulsion that facilitates the detection of small leaks and allows repetition of the test as needed. In comparison to isotonic saline, the White test has been shown to increase intraoperative bile leak detection and decrease postoperative bile leakage^13^.

Although the White test is performed by several centers worldwide, there is a need to standardize the performance of the test, especially in cases of biliary anatomical variations and in patients with previous cholecystectomy. Low insertion of the cystic duct, which precludes placement of a clamp below the confluence of the cystic duct and common hepatic duct, occurs in approximately 10% of the population^18^ ^,^ ^19^. If the cystic duct is cannulated and the clamp is not adequately positioned below its confluence with the common hepatic duct, the increased endobiliary pressure might be transmitted to the distal CBD. Since the CBD joins the pancreatic duct, forming a common duct in approximately 60% of patients^1^, the increased pressure might be transmitted to the pancreatic duct, potentially triggering acute pancreatitis. In fact, three cases of acute pancreatitis following liver resection with White test via the cystic duct in patients with low insertion of the cystic duct have been reported^5^. Moreover, previous cholecystectomy, which makes cannulation of the cystic duct stump difficult, if not impossible, is not uncommon among patients undergoing liver resection.

In the present study, we describe a technique to perform the White test that can be easily reproduced by other centers. In this technique, we emphasize the importance of clamping the CBD distal to the catheter entry site. To achieve this, whenever the cystic duct is not identified, as might be the case in patients with previous cholecystectomy, or found to insert distally in the common hepatic duct, the CBD was used for cannulation. Clamping the distal bile duct achieves two important goals: first, it allows injection of high volumes of the lipid solution in the biliary tree, which probably increases the sensitivity of the test; second, it prevents reflux to the distal CBD, a potential trigger of acute pancreatitis. In our cohort, none of the 17 patients developed clinically significant biliary leakage. Two patients developed grade A biliary leakage, with drain bilirubin concentration barely above three times as high as serum bilirubin and no change in postoperative management. If drains were not routinely left in place, those patients would probably have been asymptomatic and their grade A biliary leakage would have gone unnoticed. Additionally, despite the routine measurement of amylase and lipase, none of the patients developed acute pancreatitis, reinforcing the safety of the technique reported herein. Notwithstanding the small sample, we believe this technique to be promising for both maximizing intraoperative bile leak detection and mitigating the risk of complications associated with the test.

## CONCLUSIONS

The standardized technique for the White test, which focuses on cystic duct or CBD cannulation with distal CBD clamping, seems a safe and adequate strategy to maximize intraoperative bile leak detection and prevent acute pancreatitis.
